# Measuring the Capacitance of Carbon in Ionic Liquids: From Graphite to Graphene

**DOI:** 10.1021/acs.jpcc.3c08269

**Published:** 2024-02-21

**Authors:** Jing Yang, Athanasios A. Papaderakis, Ji Soo Roh, Ashok Keerthi, Ralph W. Adams, Mark A. Bissett, Boya Radha, Robert A. W. Dryfe

**Affiliations:** † Department of Chemistry and Henry Royce Institute, The University of Manchester, Oxford Road, M13 9PL Manchester, U.K.; ‡ Department of Materials, The University of Manchester, Oxford Road, M13 9PL Manchester, U.K.; § National Graphene Institute, The University of Manchester, Oxford Road, M13 9PL Manchester, U.K.; ∥ Department of Physics and Astronomy, The University of Manchester, Oxford Road, M13 9PL Manchester, U.K.

## Abstract

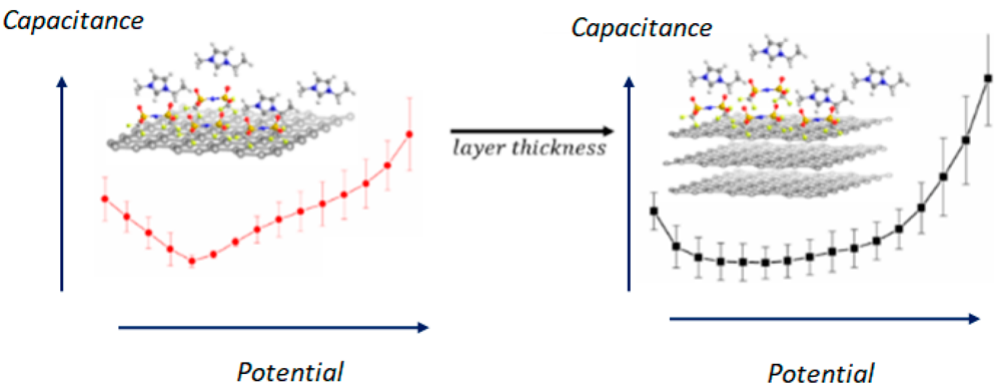

The physical electrochemistry of the carbon/ionic liquids interface underpins the processes occurring in a vast range of applications spanning electrochemical energy storage, iontronic devices, and lubrication. Elucidating the charge storage mechanisms at the carbon/electrolyte interface will lead to a better understanding of the operational principles of such systems. Herein, we probe the charge stored at the electrochemical double layer formed between model carbon systems, ranging from single-layer graphene to graphite and the ionic liquid 1-ethyl-3-methylimidazolium bis(trifluoromethylsulfonyl)imide (EMIM-TFSI). The effect of the number of graphene layers on the overall capacitance of the interface is investigated. We demonstrate that in pure EMIM-TFSI and at moderate potential biases, the electronic properties of graphene and graphite govern the overall capacitance of the interface, while the electrolyte contribution to the latter is less significant. In mixtures of EMIM-TFSI with solvents of varying relative permittivity, the complex interplay between electrolyte ions and solvent molecules is shown to influence the charge stored at the interface, which under certain conditions overcomes the effects of relative permittivity. This work provides additional experimental insights into the continuously advancing topic of electrochemical double-layer structure at the interface between room temperature ionic liquids and carbon materials.

## 1 Introduction

The development of sustainable electrochemical energy storage systems exhibiting a robust performance over many cycles while retaining fast charge storage kinetics and high efficiency is currently on the frontline of energy research. Electrochemical double-layer capacitors (EDLCs), known generally as supercapacitors, exhibit distinctive energy storage characteristics that combine fast charging/discharging kinetics with high capacity, power density, and long cycle life.^1,2^ The main drawback of EDLCs is their low energy density compared to secondary batteries. To improve the energy density of conventional EDLCs, a tailored combination of electrode and electrolyte materials with advanced physicochemical properties delicately tuned for each application is indispensable.^3,4^

Carbon-based materials are gaining increasing interest for applications as electrodes in various energy storage devices due to their unique properties such as high electronic conductivity, (electro)chemical inertness, low density, and low cost. In addition, nanostructured carbon-based materials show extremely high specific surface area (SSA) extending to the order of 500–3000 m^2^ g^–1^, hence significantly increasing the mass specific capacitance *C*_*m*_, defined as the product *C*_*A*_*A*/*m*, where *C*_*A*_ is the area-normalized capacitance (per area), *A* is the specific surface area, and *m* the mass of the active material. Note that SSA values exceeding ca. 1200–1500 m^2^ g^–1^ have no effect on both the gravimetric and area specific capacitances, which under certain conditions can even decrease above the aforementioned SSA range.^5,6^ This phenomenon, although not yet fully understood, has been suggested to be related to the low pore wall thickness in highly porous carbons. The latter is believed to decrease the density of states (DoS) near the Fermi level of carbon, which in turn limits the total capacitance of the electrode.^7^ To clarify the factors limiting the capacitance of carbon-based materials, particular focus has been given to the study of well-characterized sp^2^ carbon allotropes such as highly oriented pyrolytic graphite (HOPG) and graphene.^8^ These materials have been used as model systems to investigate the effect of the electronic properties of carbon on the total capacitance of the system.^5,9−12^

Room temperature ionic liquids (RTILs) are a class of compounds composed solely of cations and anions that exist in the liquid state at room temperature due to their relatively large ion sizes and nonuniform molecular charge density.^13^ “Solvent free” ionic liquids possess many unique physicochemical properties such as high thermal and chemical stability, low volatility, nonflammability, extreme electrochemical stability (a wide potential window of up to 5–6 V has been identified), and tunable polarity, which make them highly promising electrolytes for EDLCs. The absence of solvent in neat RTILs leads to some of the properties identified above, meaning that the theories developed to describe ionic liquids at electrified interfaces differ considerably from those relating to conventional aqueous and nonaqueous electrolytes. The “abnormal” decay length of ionic liquids near charged surfaces has been a topic of prolonged discussion, as a means to understand the role of electrostatic screening in these electrolytes in relation to the “real” Debye length.^14−19^ The structural characteristics of the EDL in ionic liquids differ significantly from the predictions of the classical Gouy–Chapman–Stern models.^20^ Capacitance profiles deviate from the traditional “U-shape” theoretically predicted and experimentally validated for a vast range of aqueous and nonaqueous electrolytes on metals. Depending on “lattice saturation” of the ionic liquids at a metallic surface, capacitance–potential plots normally exhibit “camel” or “bell” shapes.^7,21,22^ Lattice saturation of neat RTILs near an electrochemical interface reflects the free volume (voids) at the interface formed by the various impurities in the electrolyte (e.g., water introduced upon exposure to ambient conditions) and/or the uncharged chains of the ions (termed neutral voids), such as alkyl groups. Within a small to medium potential range, ions take up or replace voids, leading to an increase in ion density adjacent to the electrode and hence higher capacitance. As the applied potential bias increases, the extent of lattice saturation approaches its maximum value, resulting in a thicker EDL without inducing further image charges on the electrode. This leads to a decrease in the capacitance of the system that gives rise to a “camel” shape profile. In the case of condensed RTILs, i.e., ionic liquids with low free volume and high lattice expansion values at the interface, the decrease in capacitance with the applied potential bias is more profound even from low values of the latter, thus resulting in a “bell” shape profile.^13^ These distinct structural characteristics of the EDL in RTILs have been confirmed experimentally using metals (such as Pt^23^ and Au^24^) where the DoS is nearly infinite near the Fermi level. However, the applicability of these models on materials with finite, potential-dependent DoS such as semimetals and carbon-based materials has not been studied systematically.

In this work, we use HOPG and graphene sheets of varying thickness prepared by chemical vapor deposition on SiO_2_/Si substrates as model systems to investigate the effect of the electronic properties of carbon-based materials on the charge storage mechanisms at the EDL formed in contact with the ionic liquid 1-ethyl-3-methylimidazolium bis(trifluoromethylsulfonyl)imide (EMIM-TFSI). We demonstrate that the space charge and the quantum capacitances of the HOPG and graphene sheets, respectively, are the major factors limiting the total EDL capacitance within small to moderate potential ranges in pure EMIM-TFSI. Notably, the capacitance dependence on potential does not exhibit the common “camel” or “bell” shape and “U-shape” predicted for RTILs and aqueous electrolytes on metallic electrodes. The low DoS near the Fermi level for carbon-based electrodes results in a decreased contribution of the electrolyte to the total EDL capacitance of the interface compared to metals. The effect of electrolyte solvation is further investigated by monitoring the capacitance–potential dependence for EMIM-TFSI diluted with solvents of differing relative permittivity: the data are interpreted on the basis of the physicochemical properties of the mixtures and the structural characteristics of the EDL.

## 2 Experimental Section

### 2.1 Materials and Chemicals

Highly ordered pyrolytic graphite (HOPG) (ZYA grade, mosaic spread 0.4 ± 0.1°) was purchased from Scanwel Ltd., UK. 1-Ethyl-3-methylimidazolium chloride (≥95%), dimethyl carbonate (DEC, 99%), propylene carbonate (PC, 99.7%), chlorobenzene (anhydrous, 99.8%), and acetonitrile (ACN, 99.9%) were purchased from Sigma-Aldrich. Lithium bis(trifluoromethanesulfonyl)imide (99%) was supplied by Fluorochem. Dimethyl sulfoxide (DMSO, ≥99.5%, water content: <0.2%) and formamide (FD) (99.5%) were purchased from Thermo Fisher Scientific.

### 2.2 Synthesis of Monolayer Graphene

Monolayer graphene was synthesized by chemical vapor deposition (CVD). In detail, single-layer graphene was grown on 25 μm thick Cu foils (99.999% purity, Alfa Aesar) using a CVD furnace equipped with a quartz tube of 1 in. diameter. Cu foils were pretreated with nitric acid, acetone, and propan-2-ol prior to use, subsequently heated to 1020 °C, and annealed for 2 h under 40 sccm of hydrogen flow (99.999%). Following the annealing step, 1.5 sccm of methane (99.999%) was introduced into the tube to grow graphene. The duration of the growth process was 20 min, and upon completion, the furnace was left to cool to room temperature.

### 2.3 Preparation of Multilayer Graphene

The as-synthesized monolayer graphene on Cu was transferred to a SiO_2_/Si wafer using the general wet transfer method, assisted by a poly(methyl methacrylate) (PMMA) layer. 2% w/v of PMMA (MW 350K, Sigma-Aldrich) solution in chlorobenzene was spin-coated on the single-layer graphene/Cu samples. A 0.5 M FeCl_3_ aqueous solution was used to remove Cu, and the PMMA/graphene layer was rinsed with copious amounts of deionized water multiple times before it was collected and stacked on another graphene/Cu layer. To achieve the desired number of graphene layers (sample thickness), the above transfer process was repeated accordingly, with the PMMA/graphene layer being replaced as the substrate with a SiO_2_/Si wafer. To improve the physical contact between the individual graphene layers, after each “scoop” step, the samples were dried at 80 °C for 12 h and heated at 120 °C for 1 h. Finally, the PMMA layer was removed using acetone at room temperature.

### 2.4 Preparation of the Electrodes

HOPG and CVD graphene of varying thicknesses on SiO_2_/Si served as working electrodes. The electrical connection to HOPG was achieved by directly attaching a Cu wire (RS components, UK) at the edge of HOPG adhered with silver conductive epoxy resin (RS components, UK). Following a 24 h curing period, silver epoxy was covered by an insulating resin (Araldite) and left to dry for ca. 3 h. In the case of the CVD graphene samples, electrical connection was made in a similar way whereby the Cu wire was directly attached to the basal plane of graphene. A Pt wire (99.9% purity, 0.404 mm diameter, annealed, Alfa Aesar) was used as a counter electrode. A bipolar reference electrode (BPRE) was employed for all experiments to minimize any leakage from the reference electrode solution (i.e., water and electrochemically active ions such as Ag^+^) in the working electrolyte.^25,26^ For the preparation of the BPRE, a silver wire (99.99% purity, 0.20 mm diameter, Goodfellow Cambridge limited) was anodized in 0.5 M hydrochloric acid (Fisher Scientific) by applying three consecutive potential pulses at 0.5 1.0, and 1.5 V, each for 30 min. The procedure was conducted in a single compartment cell adopting a two-electrode configuration, where a Pt mesh served as the counter electrode. A platinum wire (0.404 mm diameter, 99.9%, Alfa Aesar) with a length of ca. 2.5 cm was sealed in a borosilicate glass tube (8 mm diameter), exposing its two ends outside and inside the tube. Subsequently, the glass tube was filled with a 3 M KCl (Sigma-Aldrich) solution, then the prepared Ag/AgCl wire was carefully inserted in the tube, and finally the upper opening was sealed with epoxy resin to prevent evaporation of the electrolyte. In all experiments, the leakless bipolar reference electrode was checked by comparing it with Ag/AgCl immersed into a saturated KCl solution to ensure the accuracy and stability of the bipolar electrode. Unless, otherwise specified, the applied potentials throughout the article are quoted vs the bipolar Ag/AgCl_(3 M KCl)_ electrode.

### 2.5 Synthesis of 1-Ethyl-3-methylimidazolium Bis(trifluoromethylsulfonyl)imide

1-Ethyl-3-methylimidazolium chloride ([EMIM]Cl) (75 g, 0.5115 mol) and lithium bis(trifluoromethylsulfonyl)imide (154 g, 0.54 mol) were dissolved in water (100 mL) in two different flasks. The two solutions were mixed under continuous stirring overnight, and the temperature of the mixture was kept at 40 °C. The resultant solution was placed in a separating funnel and left to rest until two distinct phases were formed. After complete separation, the bottom phase, consisting mainly of the ionic liquid, was carefully collected, while the top phase containing aqueous LiCl impurities was discarded. The ionic liquid phase collected during the first stage of separation (contaminated with water and LiCl impurities) was mixed with deionized water with a volume at least twice as that of the ionic liquid phase, stirred, and afterward transferred to the separating funnel. This washing process was repeated at least 10 times to ensure that LiCl impurities were completely removed. The resultant pure ionic liquid was heated at 70 °C under vacuum (<6 × 10^–2^ bar) for 3 days to remove residual water.

The synthesized ionic liquid, 1-ethyl-3-methylimidazolium bis(trifluoromethylsulfonyl)imide (EMIM-TFSI), was characterized by ^1^H, ^13^C, ^19^F, and ^7^Li NMR spectroscopy (see below and the relevant section in the Supporting Information). The water content in the EMIM-TFSI electrolyte was tested by Karl Fischer titration (Mettler Toledo V205). For pure “dried” EMIM-TFSI, the water content was found to be 2010 ppm, while after exposure to ambient conditions for 12 h, the content increased to 4090 ppm.

### 2.6 Electrochemical Measurements

**Figure 1:**
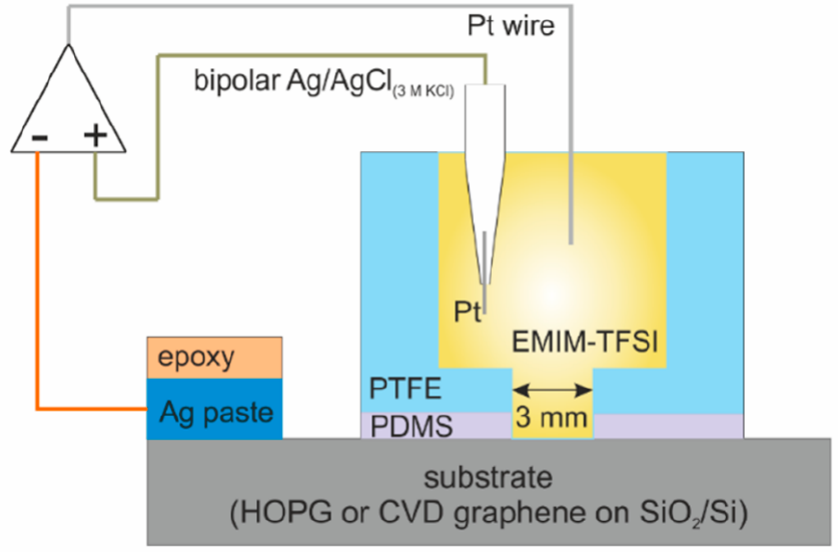
Schematic of the capacitance measurement setup.

The setup used for the capacitance measurements is described in detail in our recent work.^27^ Briefly, a hollow polytetrafluoroethylene (PTFE) cylinder (volume of ca. 0.2 cm^3^) with a disk-shaped opening of 3 mm diameter (hence an electrode nominal area of ca. 0.07 cm^2^) was placed on the basal plane of HOPG or graphene samples and used as the electrolyte container. To avoid leakage of the electrolyte, the bottom part of the cylinder was coated by a thin (ca. 1 mm) poly(dimethylsiloxane), PDMS, gel layer (Sylgard 527, Dow Corning). The setup is schematically shown in Figure 1.

All electrochemical measurements were performed on an Autolab PGSTAT302N potentiostat, equipped with a frequency response analyzer (module FRA32). Prior to each measurement, HOPG was carefully cleaved mechanically by using the “Scotch” tape method to generate a clean, fresh surface. To avoid contamination by the adsorption of airborne hydrocarbons, the PTFE cell was placed on the surface of the samples within 1 min; in the case of HOPG any visible steps and edges were avoided. Electrochemical impedance spectroscopy (EIS) was performed within the frequency range between 20 kHz and 1 Hz, with an AC amplitude of 7.07 mV rms. The dependence of EDL capacitance on the applied potential bias was probed based on the following experimental protocol: (i) The open circuit potential (OCP) of the system was determined by monitoring its variation with time until a stable value was obtained. The latter was considered for an d*E*/d*t* value of less than 1 μV/s. (ii) The OCP value was used as the starting point and the potential was stepped in increments of 100 mV initially toward the cathodic limit of the potential window of the electrolyte. (iii) After completion of step ii, the cell was left at no applied bias to reach equilibrium. An equilibrium state was confirmed by the attainment of a stable OCP value following step i. The time duration required for a stable OCP was found to vary among measurements from ca. 25 to 70 min. Subsequently, the total procedure was repeated toward the anodic limit. The use of OCP as a reference value and starting point has been reported in the literature to yield more reproducible data with ionic liquids compared to the use of an arbitrary potential bias (e.g., 0 V) often used in capacitance measurements.^23,28^

The total capacitance of the interface was extracted from the EIS data by adopting the graphical approach developed by Tribollet et al. for systems exhibiting frequency dispersion effects.^29^ An effective capacitance, *C*_eff_, is calculated at each applied frequency using the following equation:
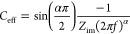
1where α is the constant phase exponent, *Z*_im_ the imaginary part of impedance, and *f* the applied frequency in Hz. The final capacitance values were obtained by averaging the determined *C*_eff_ values within the frequency range where the phase angle of the system was higher (per absolute value) than 80°.

### 2.7 Nuclear Magnetic Resonance (NMR) and Raman Spectroscopy Measurements

NMR measurements were conducted on a Bruker AVIII HD 400 (400 MHz) NMR spectrometer. A coaxial insert (GPE Scientific Ltd., Leighton Buzzard, UK, NI5CCI-B) was placed inside each standard 5 mm NMR tube, filled with a reference solvent mixture composed of 80% deuterated DMSO, 10% tetramethylsilane (TMS), and 10% triflorotoluene (TFT) to provide lock and reference signals. The electrolyte of interest was placed in the main compartment of the tube, physically separated by the reference solvents, as illustrated in Figure S1. All NMR data were collected at 298 K. ^13^C DEPTQ 135, rather than standard ^13^C{^1^H}, experiments were performed to determine ^13^C chemical shifts and the number of attached ^1^H, with maximum sensitivity.

### 2.8 Raman Spectroscopy Measurements

**Figure 2:**
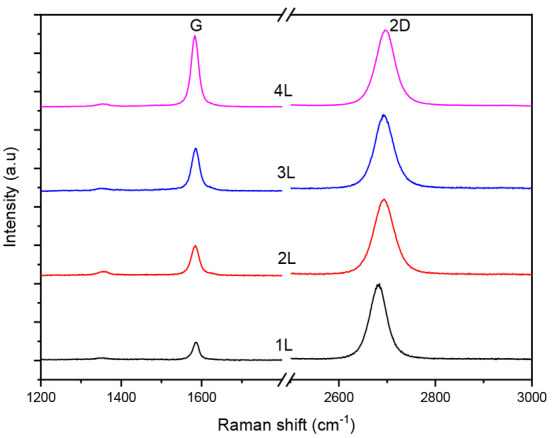
Raman spectra of the monolayer and multilayer CVD graphene.

Raman spectra were collected with 514 nm excitation and a 500× objective using a Renishaw 2000 spectrometer to confirm the layer number of the prepared graphene samples and their defect density.^30^Figure 2 shows the Raman spectra acquired for the 1- to 4-layer graphene samples. The 2D band of the monolayer is located at ca. 2682 cm^–1^ and is shifted to higher wavenumber up to ca. 2697 cm^–1^ for the 4-layer sample due to the reduced 2D_1A_ component.^31^ The G/2D band intensity ratio is ca. 0.23 for monolayer graphene, in agreement with previous studies.^32^ An increase in the same ratio compared to the monolayer is seen in the 2-, 3-, and 4-layer samples, which however is inconsistent possibly due to the random stacking order between the individual layers.^33^ A small trace in the D peak region is observed for all samples most probably originating from defects cumulated at the lowest layer due to the nature of the CVD process. The D/G band intensity ratio is equal to ca. 0.08, 0.14, 0.05, and 0.04 for 1-, 2-, 3-, and 4-layer samples, respectively, indicating the very low defect density and thus the high quality of the prepared graphene samples.

## 3 Results and Discussion

The charging mechanisms of ionic liquids at electrodes with finite DoS near the Fermi level have not been studied systematically from an experimental point of view. On this basis, we start by investigating the capacitance of the HOPG | neat EMIM-TFSI (ca. 3.89 M) interface. The basal plane of HOPG is an almost ideal model carbon surface, exhibiting uniform sp^2^ hybridization with an atomically smooth morphology. The free electron density of HOPG is ca. 8.6 × 10^18^ cm^–3^ (298 K),^34^ a value orders of magnitude less than that in metals (e.g., 6 × 10^22^ cm^–3^ for Au) and other carbon materials such as glassy carbon (2 × 10^20^ cm^–3^).^7^ This limited DoS near the Fermi level gives rise to an additional potential drop at the interface distinct from that associated with the Galvani potential. This additional contribution occurs within the solid and is typically referred as space-charge capacitance or quantum capacitance for two-dimensional electrodes such as graphene.^35^ The total capacitance, *C*, of these systems can be expressed as^35^
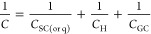
2where *C*_SC__(or q)_ is the space charge (or quantum) capacitance, *C*_H_ the Helmholtz capacitance, and *C*_GC_ the capacitance associated with the Gouy–Chapman (or diffuse) layer. The sum 1/*C*_H_ + 1/*C*_GC_ can be defined as the capacitance associated with the electrolyte side (i.e., the EDL), 1/*C*_EDL_. For high electrolyte concentrations (typically above 0.1 M), *C*_GC_ becomes larger and its contribution to *C* decreases with electrolyte concentration. In this case, *C* is governed by *C*_H_ and *C*_SC__(or q)_.^20^

**Figure 3:**
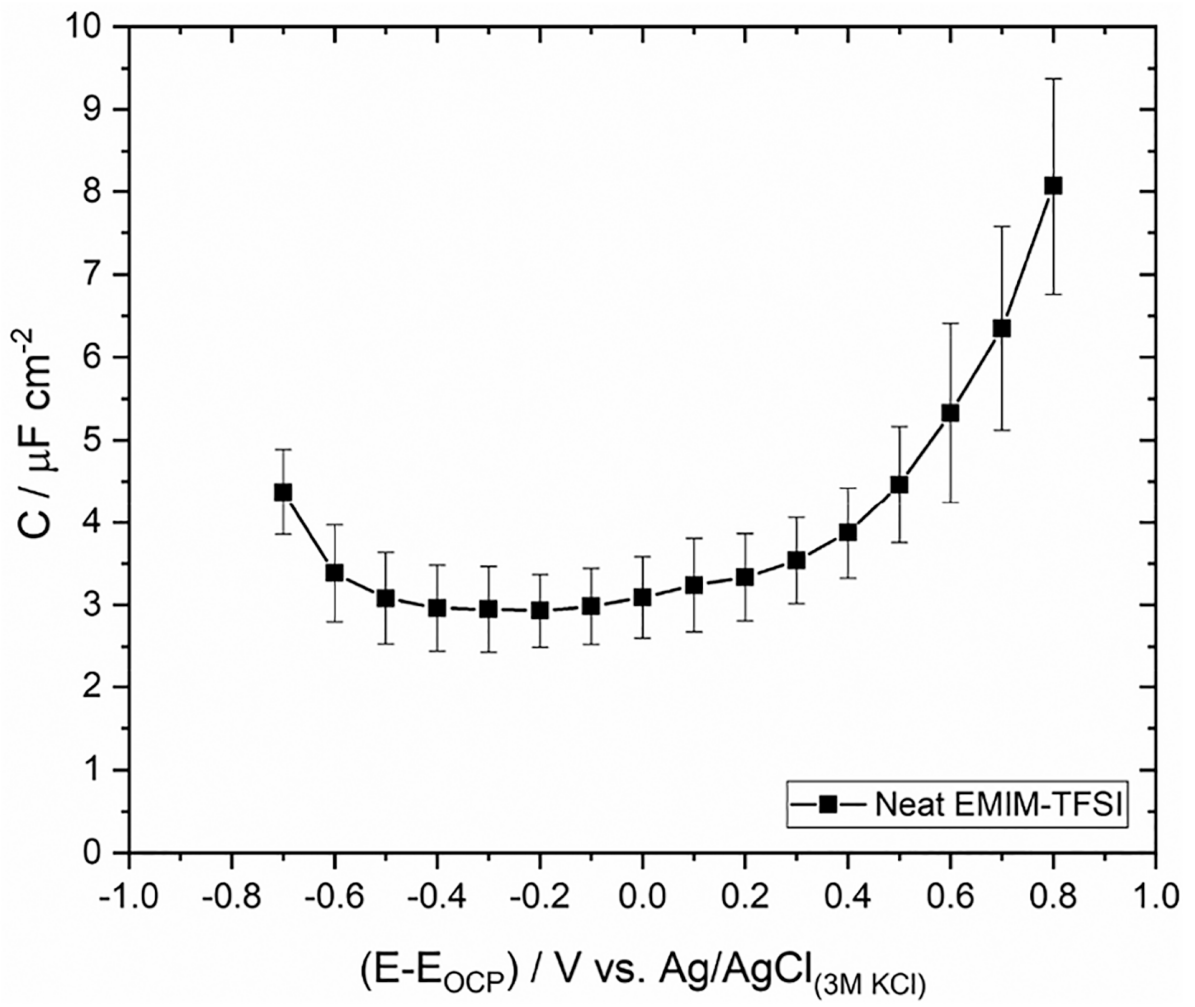
Differential capacitance, *C*, vs applied potential, *E*, plot recorded at the HOPG|neat EMIM-TFSI interface. *C* was extracted using eq 2 from the EIS data recorded in the capacitive potential window (see Experimental Section). The potential window of the electrolyte was estimated by the phase angle between the AC voltage and current in the corresponding Bode phase plots (Figure S4).

Figure 3 shows the differential capacitance of the HOPG|neat EMIM-TFSI interface obtained using EIS and following the protocol described in the Experimental Section. The data were recorded within the potential window of the electrolyte (ca. −0.7 to 0.8 V vs Ag/AgCl_(3 M KCl)_) which has been estimated by monitoring the phase angle between the applied AC voltage and current in the corresponding EIS Bode phase plots (see Figure S4). Outside of this region, faradaic processes related to the interaction of water impurities (ca. 0.2%; refer to the Experimental Section) with HOPG occur. The hydrophobic character of EMIM-TFSI drives the water molecules toward the electrode surface compared to more hydrophilic ionic liquids, such as 1-butyl-3-methylimidazolium trifluoromethanesulfonate (BMIM-OTf), where water is adsorbed into the bulk region. Consequently, the potential window of hydrophobic ionic liquids in the presence of water impurities is often reported to be narrower than that of hydrophilic ones.^36,37^ The first, notable qualitative characteristic of the *C* vs *E* plot is its “U-like shape”. The minimum in *C*, denoted as *C*_min_, equal to ca. 3.1 μF cm^–2^ extends over a potential region of ca. 500 mV. This value is very close to that reported for HOPG in 1-butyl-3-methylimidazolium tetrafluoroborate (BMIM-BF_4_).^9^ Interestingly, the minimum capacitance reported for the same material in various aqueous and nonaqueous electrolytes within a wide range of concentrations shows very similar values. In more detail, *C*_min_ is found to lie within ca. 2.7–2.9 μF cm^–2^ in aqueous NaF solutions of 10^–2^–0.9 M,^38^ ca. 2.5 μF cm^–2^ in aqueous solutions of KF in the concentration range between 0.1 and 16 *m* (mol kg^–1^),^27^ ca. 2.8 μF cm^–2^ in 20 *m* CsF,^27^ ca. 3 μF cm^–2^ in 20 *m* lithium bis(trifluoromethanesulfonyl)imide (LiTFSI) water-in-salt electrolytes,^39^ ca. 3.2 μF cm^–2^ in 1 M LiCl in propylene carbonate,^39^ and ca. 3 μF cm^–2^ in 0.2 M tetrapropylammonium tetrafluoroborate (TPABF_4_) in acetonitrile.^34^ Considering that by definition *C*_min_ corresponds to the potential of zero charge, *E*_pzc_,^20,38^ these minor differences (of the order of hundreds of nF cm^–2^) imply that the contribution of *C*_EDL_ to *C* within this relatively narrow potential window (ca. 500 mV) is minimal. Furthermore, works on metallic electrodes report *C*_min_ to be at least double those on HOPG. For example, *C*_min_ in EMIM-TFSI on Hg^40^ and Pt^23^ are found to be 11.7 and 6.6 μF cm^–2^, respectively, with the characteristic “camel” or “bell” shapes of the *C* vs *E* plots.^13,23,24,41^ Both the low *C*_min_ values and “U-like shape” of the *C* vs *E* plots demonstrate that the low DoS of HOPG governs the total capacitance of the interface through its influence on *C*_SC_, and therefore the identity of the electrolyte has a less significant effect. Finally, the flattened “U-shape” in the capacitance plot of Figure 3 can be attributed to the strong imidazolium cation−π interaction between EMIM^+^ ions and HOPG.^42,43^ At this point, it needs to be emphasized that an accurate determination or even a sensible estimation of *E*_pzc_ based on the minimum in the *C* vs *E* plots (following the classical Gouy–Chapman theory) is not feasible. Even though *E*_pzc_ values are often reported in the literature for electrodes with finite DoS (e.g., graphite) by quoting the mathematical minimum in the *C* vs *E* plots, these values have little (if any) physical significance because the total capacitance of the interface (recorded using common electrochemical techniques) can be dominated by the solid side in the vicinity of *E*_pzc_ and hence mask the response of the electrolyte (depicted in an apparent plateau rather than a distinct minimum in the *C* vs *E* plots). The same can also result from complex interactions between the electrolyte and the electrode (see the discussion above).

Following our findings on HOPG, we aim to systematically explore the contribution of the electrode side to the total capacitance of the system by probing the effect of the changes in the electronic structure of graphene sheets with the number of layers on *C*, in neat EMIM-TFSI. Monolayer graphene is a zero-band-gap semiconductor, in contrast to graphite which exhibits a semimetallic behavior with a band overlap of ca. 41 meV. Adding another layer of graphene transforms the Dirac-like spectrum around the Fermi energy of the monolayer to a parabolic shape with a small band overlap for the bilayer (ca. 1.6 meV).^44^ Starting from 3-layer graphene and moving progressively to graphite (ca. 10–11 layers), multilayers behave as semimetals due to the interactions between the *B* carbon atoms of next-nearest-neighbor planes. In general, the increase in the number of layers increases the band overlap and hence the DoS near the Fermi level.^44,45^ On this basis, by studying the capacitance of the single-layer and multilayer graphene|neat EMIM-TFSI interfaces, we can indirectly infer the effect of the DoS (hence the electronic properties of the electrode) on the total capacitance of the system, while the electrolyte concentration and identity are kept constant.

**Figure 4:**
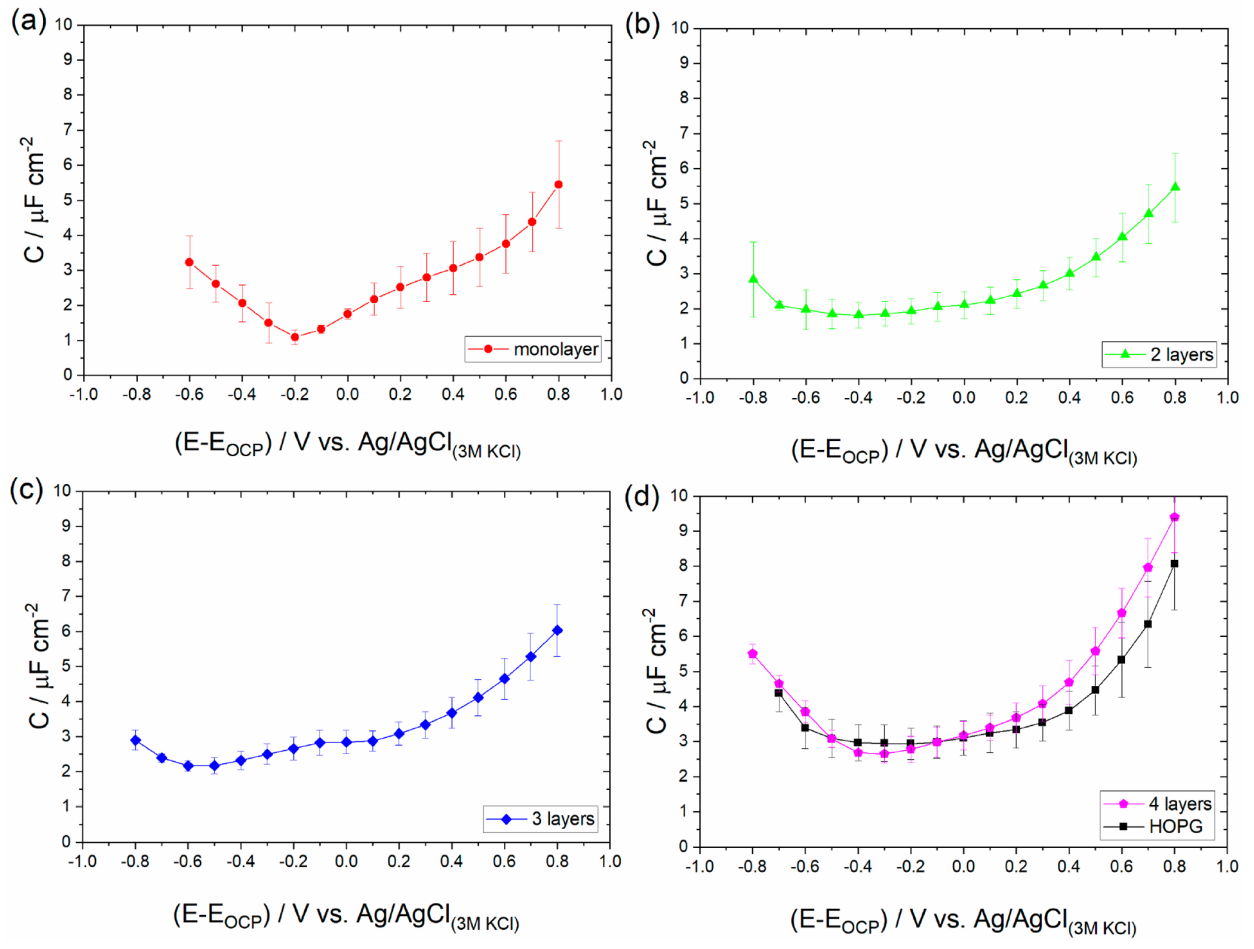
Differential capacitance, *C*, vs applied potential, *E*, plots recorded at the (a) monolayer, (b) 2-layer, (c) 3-layer, and (d) 4-layer CVD graphene sheets on SiO_2_/Si in neat EMIM-TFSI following the experimental protocol described in the Experimental Section. *C* was extracted using eq 2 from the EIS data recorded in the capacitive potential window (see Experimental Section). The data correspond to the potential window of the electrolyte, as was estimated by the phase angle between the AC voltage and current in the corresponding Bode phase plots (see Figure S5). For comparison purposes in (d), the data presented in Figure 3 corresponding to HOPG are also given.

Figure 4a presents the *C* vs *E* plot of a monolayer CVD graphene sheet on SiO_2_/Si in neat EMIM-TFSI within the potential window of the electrolyte. The latter was estimated to be ca. 1.4 V following the EIS approach described previously for HOPG. Values of 2 and 1.7 V are reported for the same ionic liquid with 0 and 33% relative humidity, respectively, using a single-layer graphene electrode and cyclic voltammetry (CV).^46^ We ascribe the lower value determined in our work to the technique used (i.e., EIS) for estimation of the potential window of the electrolyte. Charge transfer reactions (including pseudocapacitive processes) can be easily captured by EIS through deviations from the ideal capacitive response in both Nyquist and Bode plots. In particular, Bode phase plots are very sensitive to faradaic leakages arising by charge transfer processes occurring even at low rates. These are identified in the Bode phase plots as deviations in the phase angle from 90° (the latter corresponds to an ideally polarizable interface). Therefore, in contrast to the arbitrary choice of a current density value in CV experiments to define the potential window of the electrolyte (often leading to an overestimation of the electrolyte stability window), the EIS approach is significantly more sensitive because it considers (and hence rules out) both electrolyte degradation reactions (i.e., electrolysis) and pseudocapacitive processes, such as specific adsorption, which are common in ionic liquids (see e.g. ref (46)). Returning to the *C* vs *E* plot, a typical “V-shape” capacitance profile is recorded, in line with experimental data previously reported in ionic liquids.^10−12,46,47^ The characteristic shape of the *C*_EDL_ vs *E* plot is attributed to the known linear dependence of the DoS on the applied potential bias in graphene.^43,48,49^*E*_pzc_, approximated by the minimum in the *C* vs *E* plot, is located at −0.2 V, indicating the n-doping state of graphene attributed to the interaction between graphene and the TFSI^–^ anion as previously reported.^46^ However, this effect is expected to be relatively weak considering also the simultaneous combined p-doping from water impurities and SiO_2_/Si.^50,51^*C*_min_ is found to be ca. 1.1 μF cm^–2^, a value being significantly lower (by a factor of ca. 5–12) compared to earlier reports dealing with similar systems.^10,11,47^ We attribute these differences to the increased density of lattice defects, the high concentration of charged impurities (both arising by the various fabrication processes), and/or the effect of the substrate on the electronic properties of the graphene overlayer (e.g., Au in refs (11 and 47)) in the reports cited above. We postulate that these effects are the primary reason for the rather scattered experimental capacitance data found in the literature and the consequent apparent discrepancies between theory and experiment. Very recently, Zheng et al.^46^ presented a thorough investigation of the effect of water in ionic liquids on *C*_EDL_ where among other particularly interesting results, they show that *C*_min_ of CVD graphene on SiO_2_/Si substrates lies between ca. 1 and 2 μF cm^–2^ for a series of hydrophilic and hydrophobic ionic liquids (ca. 1.7 μF cm^–2^ for EMIM-TFSI). Theoretically, the DoS of pristine graphene approaches zero at 0 K,^48^ and the quantum capacitance, *C*_q_, is predicted to be ca. 0.56 μF cm^–2^ at 300 K.^43^ However, in real systems, the previously mentioned factors increase *C*_q_ (often significantly)^52^ leading to values ranging between 0.86 and 6 μF cm^–2^.^5,10,12,49,53^ Because the potential bias at which *C*_min_ is obtained defines experimentally *E*_pzc_, we expect that the former should approximate *C*_q_ because the latter is significantly lower compared to *C*_H_ due to the zero net surface charge and hence dominates the total capacitance of the system. Consequently, the *C*_min_ value determined in our study (i.e., 1.1 μF cm^–2^), being very close to the predicted *C*_q_ for monolayer graphene, demonstrates (i) the high quality of the prepared graphene samples, and most importantly (ii) it directly links our experiments with the theoretical models developed through which *C*_q_ is extracted at the single-layer graphene|ionic liquid interface.

An additional interesting characteristic in Figure 4a is the small hump in the positive region of the capacitance–potential plot. This feature is reminiscent of the dielectric saturation phenomenon frequently identified in the traditional tensametry experiments on various metals attributed to the reorientation of the solvent molecules inside the Helmholtz layer.^54^ The latter results in a local increase in the relative permittivity, which in turn increases the capacitance. Budkov et al.^55^ have predicted similar effects for ionic liquids in the case where water impurities are present in the electrolyte. Considering the hydrophobic character of EMIM-TFSI which leads to the accumulation of water molecules in the immediate vicinity of the electrode (refer to the first part within this section), it is reasonable to assume that these effects are responsible for the observed capacitance hump. A similar finding has been also reported recently by Zheng et al. for a series of ionic liquids.^46^

Moving on to investigate the effect of the number of graphene layers on *C*, we start with bilayer graphene. It can be seen from Figure 4b that even a single additional graphene overlayer flattens the capacitance profile at potentials adjacent to *E*_pzc_, while at the same time an increase in *C*_min_ (taken as the average of the values within the capacitive plateau, i.e., between −0.6 and 0.1 V) by a factor of ca. 1.8 is observed. This finding can be attributed to the increase in the DoS near the Fermi level of graphene with increasing thickness, being already evident in 2-layer graphene sheets.^44^ The transition of the capacitive profile toward a “U-like shape” (similar to HOPG) stems from the fact that the increase in DoS induces a higher density of image charges at the outmost layers,^5,56,57^ which promotes the interactions between the electrode and the solution components (both solvent molecules and electrolyte ions). Further increase in the number of graphene overlayers (Figure 4c,d) leads to similar capacitance profiles to those seen on bilayer samples, where however a gradual increase in average *C*_min_ is recorded up to the limit of the 4-layer samples: in the latter case, the average *C*_min_ practically coincides with that recorded on HOPG. This increase in *C*_min_ is interpreted on the basis of the well-established continuously enriched DoS near the Fermi level of graphene with increasing number of overlayers.^44,45^ The slightly higher *C* values recorded in the positive extremes of the applied potential window on 4-layer graphene compared to HOPG may be related to the intrinsically higher density of defects on multilayer CVD graphene,^58^ which promotes the interaction of both water and electrolyte ions with the electrode.

Having characterized the effect of the electronic properties of graphene and graphite on their total capacitance in contact with neat EMIM-TFSI, we turn to investigating the contributions of the electrolyte to *C*. To elucidate this, we used a series of solvents with different relative permittivity, ε_r_, values (see Table 1) to dilute the neat EMIM-TFSI. The aim is to probe the ability of a solvent to overcome the relatively strong intermolecular forces existing between cations and anions in ionic liquids and hence influence its dissociation degree (ca. 0.7–0.8 for EMIM-TFSI).^59^ The physicochemical relation between the capacitance and dissociation degree of an ionic liquid has been identified previously and is a topic of recurring interest.^14−16,59,60^ In general, the dissociation degree of ionic liquids is dependent on temperature, *T* (it increases with *T*), and the identity of the solvent used for dilution.^59,61^ Normally, ε_r_ can provide an approximate guide to the ability of a solvent to effectively dissociate an electrolyte, with solvents of high ε_r_ values increasing the dissociation degree. In this respect, a higher dissociation degree should increase the concentration of “free ions” inside the EDL promoting their interactions with the electrode and consequently influencing the charge mechanisms and structure of the EDL.^13^ Furthermore, the coexistence of solvent molecules and ions inside the EDL is also expected to influence its structural characteristics.

**Table 1:** Relative Permittivity at Room Temperature of the Solvents Used to Dilute the Neat EMIM-TFSI

solvent	diethyl carbonate (DEC)	acetonitrile (ACN)	dimethyl sulfoxide (DMSO)	propylene carbonate (PC)	formamide(FD)
relative permittivity (ε_r_)	3.1	38	47	64	111

**Figure 5:**
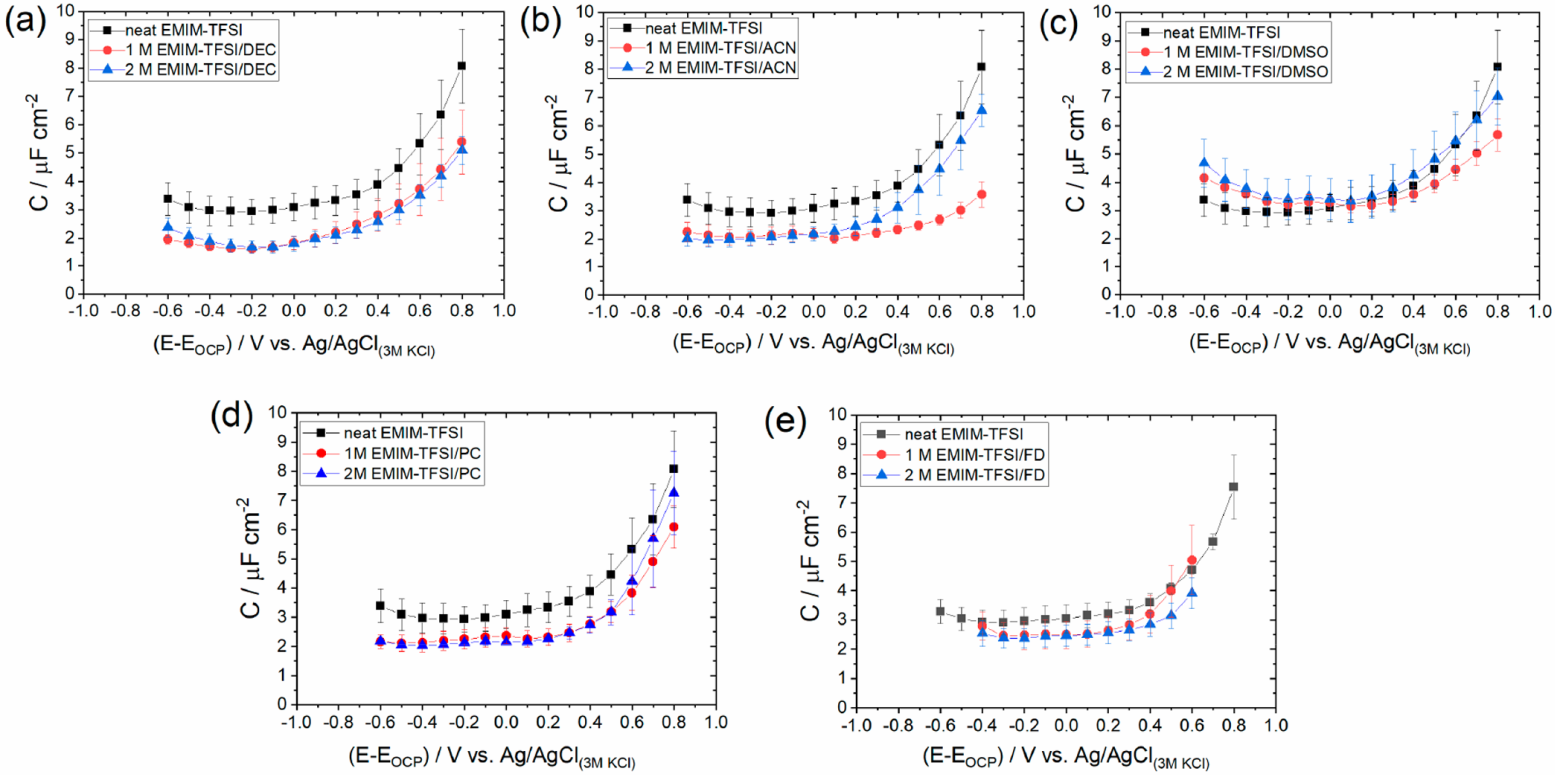
Differential capacitance, *C*, vs applied potential, *E*, plots recorded on the HOPG in contact with 1 and 2 M mixtures of EMIM-TFSI with (a) diethyl carbonate (DEC, ε_r_ = 3.1), (b) acetonitrile (ACN, ε_r_ = 38), (c) dimethyl sulfoxide (DMSO, ε_r_ = 47), (d) propylene carbonate (PC, ε_r_ = 64), and (e) formamide (FD, ε_r_ = 111) following the experimental protocol described in the Experimental Section. *C* was extracted using eq 2 from the EIS data recorded in the capacitive potential window (see the Experimental Section). The data correspond to the potential window of the electrolyte, as that estimated by the phase angle between the AC voltage and current in the corresponding Bode phase plots (see Figure S4). For comparison purposes, the data presented in Figure 3 corresponding to the neat EMIM-TFSI are also given.

**Figure 6:**
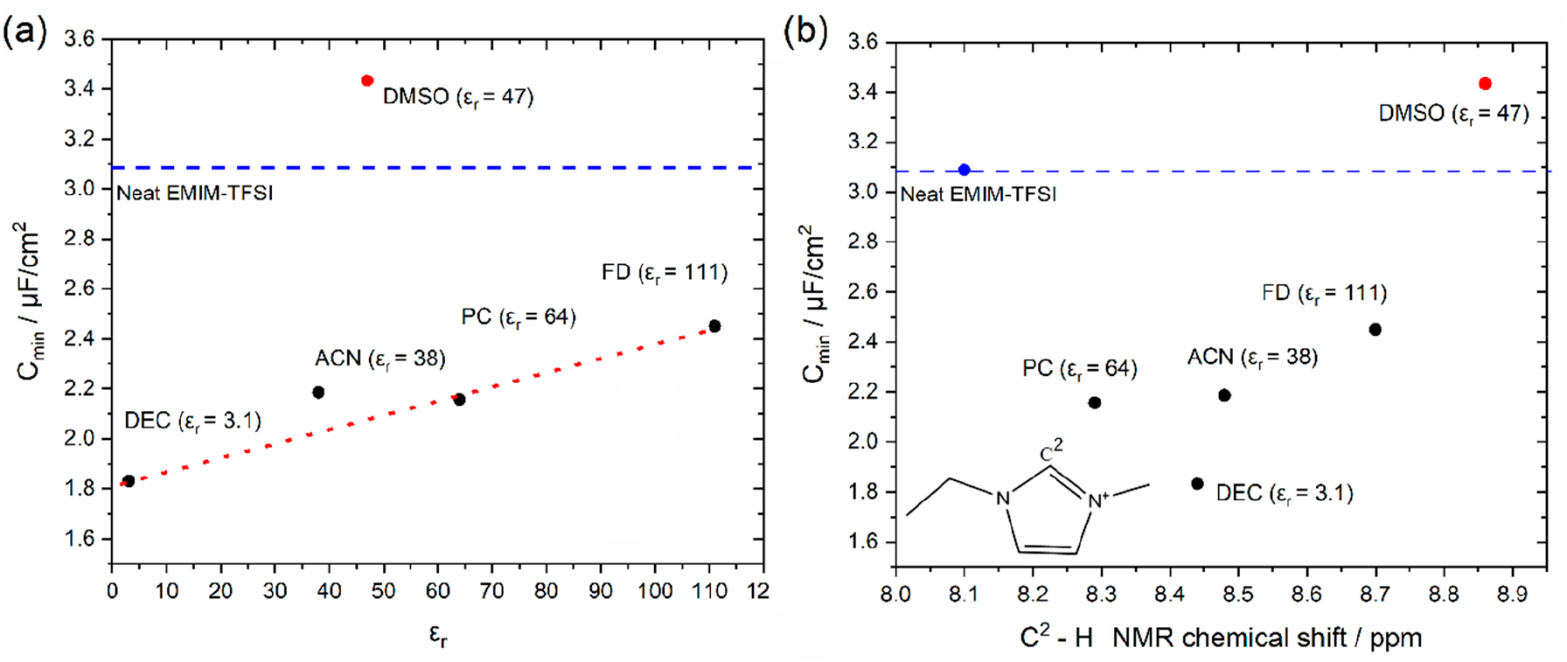
Dependence of the minimum capacitance, *C*_min_, determined by averaging the values lying within the potential region of the capacitive plateau in Figure 5, i.e., −0.3 to 0.2 V, on (a) the relative permittivity, ε_r_, of the solvents used to dilute the neat EMIM-TFSI and (b) the ^1^H NMR chemical shift corresponding to the hydrogen bonded to C^2^ in the structure of the [EMIM]^+^ cation (see the inset). The horizontal blue dotted line shows the *C*_min_ value for neat EMIM-TFSI obtained from the data presented in Figure 3. For clarity, the values of ε_r_ for all solvents are given in parentheses.

Figure 5 presents the capacitance data recorded on the basal plane of HOPG in contact with 1 and 2 M mixtures of EMIM-TFSI with a series of solvents of varying ε_r_. It is evident that apart from the EMIM-TFSI/DMSO mixture, all other mixtures exhibit on average lower *C* values compared to the neat EMIM-TFSI within the whole applied potential range. The overall decrease in *C* is reasonable because the ion density inside the EDL decreases upon dilution. Furthermore, as depicted in Figure 6a, an almost linear yet subtle increase of *C*_min_ (once again taken as the average of the constant region in the *C* vs *E* plots) is observed as ε_r_ increases, with the DMSO mixture being the outlier. This apparent increase in *C*_min_ with ε_r_ possibly indicates the more effective dissociation of the electrolyte when using solvents of higher ε_r_, a phenomenon leading to a higher density of “free ions” inside the EDL. However, in the case of the EMIM-TFSI/DMSO mixture, the above reasoning does not seem to apply, implying that additional processes are at play. Because of their complex structure, ions in ionic liquids cannot be considered as point charges. The stereochemical configuration of anions and cations gives rise to short-range intermolecular forces (such as dipole–dipole interactions and hydrogen bonding) between the ions themselves and the ions with the solvent molecules. In this respect, a sensible starting point to decipher the observed behavior in the DMSO mixtures would be to probe the intermolecular forces between the solvent molecules and the electrolyte ions. To achieve this, we studied the physicochemical properties of the prepared mixtures by employing NMR spectroscopy.

**Figure 7:**
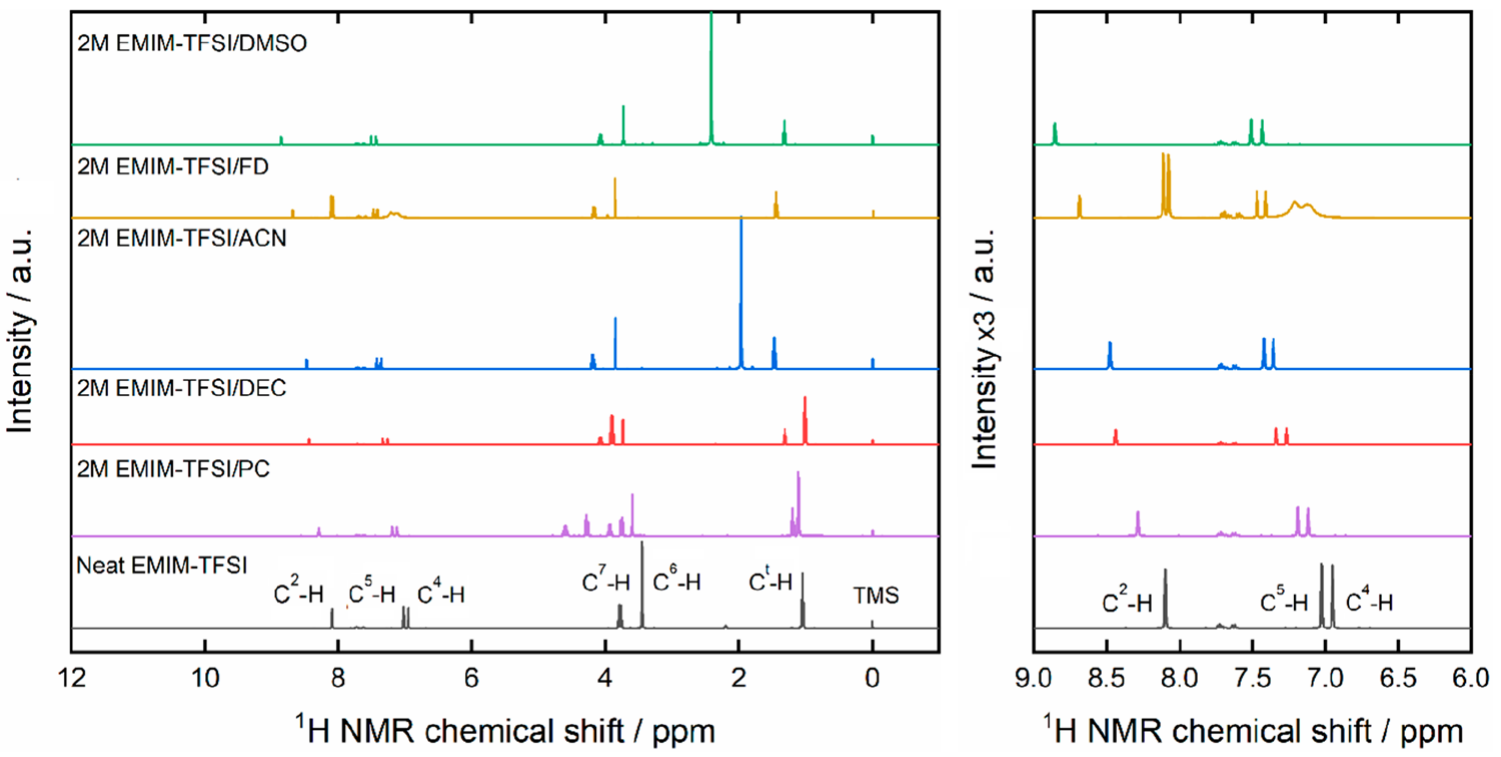
^1^H NMR spectra for the 2 M EMIM-TFSI mixtures with propylene carbonate (PC), diethyl carbonate (DEC), acetonitrile (ACN), formamide (FD), and dimethyl sulfoxide (DMSO). On the right panel, a magnification (intensity units multiplied by a factor of 3) of the chemical shift region where C^2^, C^4^, and C^5^ (see Figure S2) respond is given.

Figure 7 shows the ^1^H NMR spectra obtained for neat EMIM-TFSI and the various 2 M mixtures studied. To identify the numbers corresponding to the carbon atoms in the [EMIM]^+^ cation structure, the reader is referred to Figure S2. The ^1^H NMR spectra of the neat EMIM-TFSI shows a downfield shift of the H atom bonded to C^2^. The more electronegative N atoms bound to C^2^ in the imidazolium ring of [EMIM]^+^ induce a partial positive charge on carbon that results in a reduced electron density in the H atom connected to C^2^. A similar deshielding effect is also identified for C^4^–H and C^5^–H, though it appears to be weaker than that in the C^2^–H bond, as the C^4^ and C^5^ atoms are only directly bound to one N atom each. Compared to neat EMIM-TFSI, the peaks attributed to the H atom in the C^2^–H bond in all mixtures are downshifted, a phenomenon that demonstrates the effective solvation of the electrolyte ions (hence the increase in the degree of dissociation of the ionic liquid) by the solvents used.

Figure 6b shows the evolution of *C*_min_ with ^1^H NMR shifts detected for the H atom of the C^2^–H bond in the imidazolium ring. The former is chosen over those of C^4^–H and C^5^–H due to its stronger deshielding effect arising from it bearing the highest positive charge of the carbons in the imidazolium ring.^62^ A particular noteworthy feature in this figure is that both the largest ^1^H NMR shift (ca. 0.8 ppm) and highest *C*_min_ values (ca. 3.4 μF cm^–2^) are recorded for the EMIM-TFSI/DMSO mixture. This finding implies that the strong deshielding effect between the H atom of C^2^–H and DMSO leads to the most effective dissociation of the electrolyte in this mixture, therefore increasing the ionic density in the vicinity of the electrode. Also, it is worth noting that the capacitive plateau in Figure 5c is shifted upward compared to the pure EMIM-TFSI. Considering that within this potential range strong interactions between EMIM^+^ and HOPG are expected (see the discussion of Figure 3), the shift in capacitance further highlights the promoting effect of DMSO. The strong deshielding effect observed for the DMSO mixture is attributed to the hydrogen bonding of DMSO with the three sites of the H atoms in the imidazolium ring (i.e., C^2^, C^4^, and C^5^). In particular, the highest positive charge in the ring of the [EMIM]^+^ cation located (at the C^2^ atom) attracts the O atom in DMSO via long-range interactions:^62^ this results in the largest ^1^H NMR shift for C^2^–H among all solvents. For the mixtures of EMIM-TFSI with DEC, ACN, and FD, the dependence of *C*_min_ on ^1^H NMR shift is very similar to that discussed previously based on the relative permittivity of these solvents (Figure 6a); that is, a stronger shift leads to higher *C*_min_ with an almost linear relation among them. However, the EMIM-TFSI/PC mixture appears to deviate from this trend, exhibiting a weaker shift in the solvent series studied. Takamuku et al.^63^ have previously reported the relative weak dissociation properties of PC for imidazolium-based ionic liquids, where they also highlighted the same weak ^1^H NMR shift. The latter can be ascribed to the tail to head formation of hydrogen bonds between similar PC molecules.^63,64^

Based on the above findings, it is evident that in contrast to the general view, relative permittivity can only provide a rough estimation about the dissociation properties of a solvent when mixed with ionic liquids. Intermolecular forces between the ions constituting the ionic liquid and the solvent molecules may overcome the effects of relative permittivity and strongly influence the degree of dissociation of the electrolyte. As a result, special consideration should be given to both the physical properties of the solvents and the intermolecular forces in the mixtures. On this basis, each case should be examined independently to provide accurate insights into the complete physiochemical properties of the mixtures and their subsequent effect on the structural characteristics of the EDL in contact with various electrodes.

An additional important point is the effect of water impurities in the EMIM-TFSI/DMSO mixture, introduced due to the known hygroscopic nature of DMSO (note that the water content in pure as-received DMSO used in this study was found to be 2940 ppm by Karl Fischer titration).^65^ An increase in the amount of water compared to the rest of the mixtures could influence the chemical composition inside the Helmholtz plane (especially due to the hydrophobic nature of EMIM-TFSI; see the relevant discussion in the beginning of this section) and hence the structural characteristics of the EDL. Figure S8 shows the capacitance data recorded in EMIM-TFSI mixed with solutions of DMSO/water in two different compositions. It is evident that in both systems, *C*_min_ lies below that obtained for neat EMIM-TFSI, thus demonstrating the deleterious effect of water on *C*_min_. This is further highlighted at the positive limits of the applied potential window (above ca. +0.3 V), where the increase in water content suppresses the capacitance values, compared to the neat EMIM-TFSI. This negative effect of water on the total capacitance of the interface may be attributed to the increased density of the highly polar solvents molecules inside the Helmholtz layer (possibly found as both individual molecules and DMSO–water pairs due to the strong hydrogen bonding among them^66−68^), which increases the distance between the ions and the electrode. Overall, we can conclude that the observed increase in *C*_min_ in the EMIM-TFSI/DMSO mixture is related to the intermolecular forces between [EMIM]^+^ and the DMSO molecules, as previously discussed.

## 4 Conclusions

A systematic study of the structural characteristics of the electrochemical double layer, formed between model carbon systems ranging from single-layer graphene to graphite and the ionic liquid EMIM-TFSI, is presented. The strong effect of the electronic properties of the electrodes on the total capacitance of the interface within a small to medium potential range was demonstrated, and its dominance to the charging mechanisms of the system over that arising by the electrolyte side in pure ionic liquid is highlighted. In mixtures of the ionic liquid with various solvents, the capacitance of the interface is shown to be dependent on both the relative permittivity of the solvent and the intermolecular forces between the electrolyte ions and the solvent molecules. This is highlighted in the case of mixtures with DMSO, where the hydrogen bonding between DMSO molecules and the imidazolium ring in [EMIM]^+^ cation increases the dissociation degree of the ionic liquid leading to higher capacitance values, compared to pure ionic liquid and its mixtures with solvents of even higher relative permittivity, by overcoming the effects of the latter. The introduced general strategy based on the intermolecular interplay between the electrolyte ions and the solvent molecules is envisaged to be applicable to various electrodes for the interpretation of the capacitive response in dilute ionic liquids. Overall, our findings can have direct implications on the mechanistic studies of charge storage at the carbon/ionic liquid interface, therefore being relevant to the performance of supercapacitors operating in ionic liquid-based electrolytes. Furthermore, the reported physicochemical insights into the graphite–electrolyte ion interactions in diluted ionic liquids can be applied to the studies of ion intercalation into graphite in similar electrolyte environments from the perspective of the interplay between the solvation and intercalation energies.

## Supporting Information Available

^1^H, ^13^C,^19^F, and ^7^Li NMR spectra of neat EMIM-TFSI and additional experimental details; electrochemical impedance spectra for the neat and diluted EMIM-TFSI; ^13^C and ^19^F NMR spectra of the EMIM-TFSI mixtures; capacitance data for the EMIM-TFSI/DMSO/H_2_O mixtures (PDF)
