# Supplementary material for: Measuring the Capacitance of Carbon in Ionic Liquids: From Graphite to Graphene
Source: J Phys Chem C Nanomater Interfaces. 2024 Feb 21;128(9):3674–84. doi: 10.1021/acs.jpcc.3c08269 (PMC10926162; doi:10.1021/acs.jpcc.3c08269)
Supplement: Supplementary file 1 — jp3c08269_si_001.pdf [file jp3c08269_si_001.pdf]

Supporting Information

Measuring the Capacitance of Carbon in Ionic Liquids: From Graphite to Graphene

Jing Yang[1], Athanasios A. Papaderakis[1], Ji Soo Roh[2,3], Ashok Keerthi[1,3], Ralph W. Adams[1], Mark A. Bissett[2,3], Boya Radha[4], Robert A.W. Dryfe[1 *]*

[1]Department of Chemistry and Henry Royce Institute, The University of Manchester, Oxford Road, M13 9PL, Manchester (UK)

[2]Department of Materials, The University of Manchester, Oxford Road, M13 9PL, Manchester (UK)

[3]National Graphene Institute, The University of Manchester, Oxford Road, M13 9PL, Manchester (UK)

[4]Department of Physics and Astronomy, The University of Manchester, Oxford Road, M13 9PL, Manchester (UK)

Email: athanasios.papaderakis@manchester.ac.uk (A.A.P.), robert.dryfe@manchester.ac.uk (R.A.W.D.)

1. ^{1}H, ^{13}C,^{19}F and ^{7}Li NMR spectra of neat EMIM-TFSI

As mentioned in the main text, the internal NMR shift solvents composed of 80% (by volume) deuterated-DMSO, 10% tetramethylsilane (TMS) and 10% triflorotoluene (TFT). The electrolyte of interest was placed in the main compartment of the tube, physically separated by the reference solvents as illustrated in Figure S1.

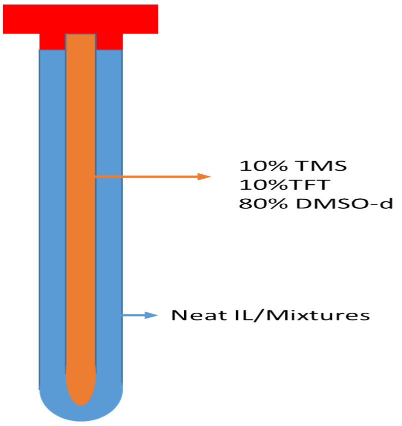

Figure S1: Internal NMR reference solvents and measured solution setup.

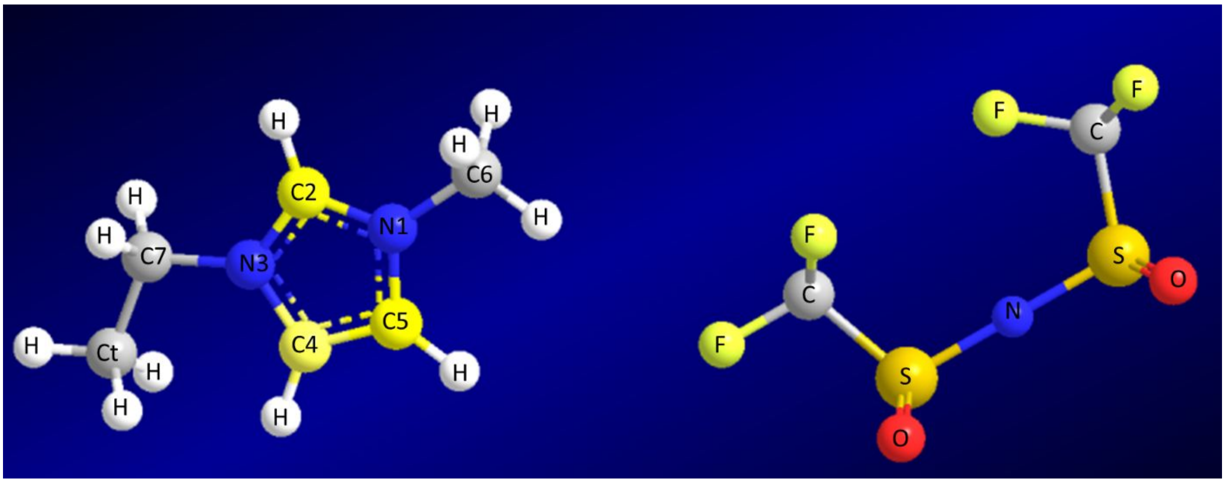

Figure S2: Chemical structure of EMIM-TFSI.

^{1}H, ^{13}C and ^{19}F NMR spectra of neat EMIM-TFSI are shown in Figure S3. The recorded chemical shifts are consistent with the literature[1] validating the successful synthesis of the ionic liquid. The absence of peaks in the ^{7}Li NMR spectrum (not shown, the spectrum was essentially noise) demonstrates the complete removal of LiCl during the synthesis of EMIM-TFSI (see Experimental section in the main text).

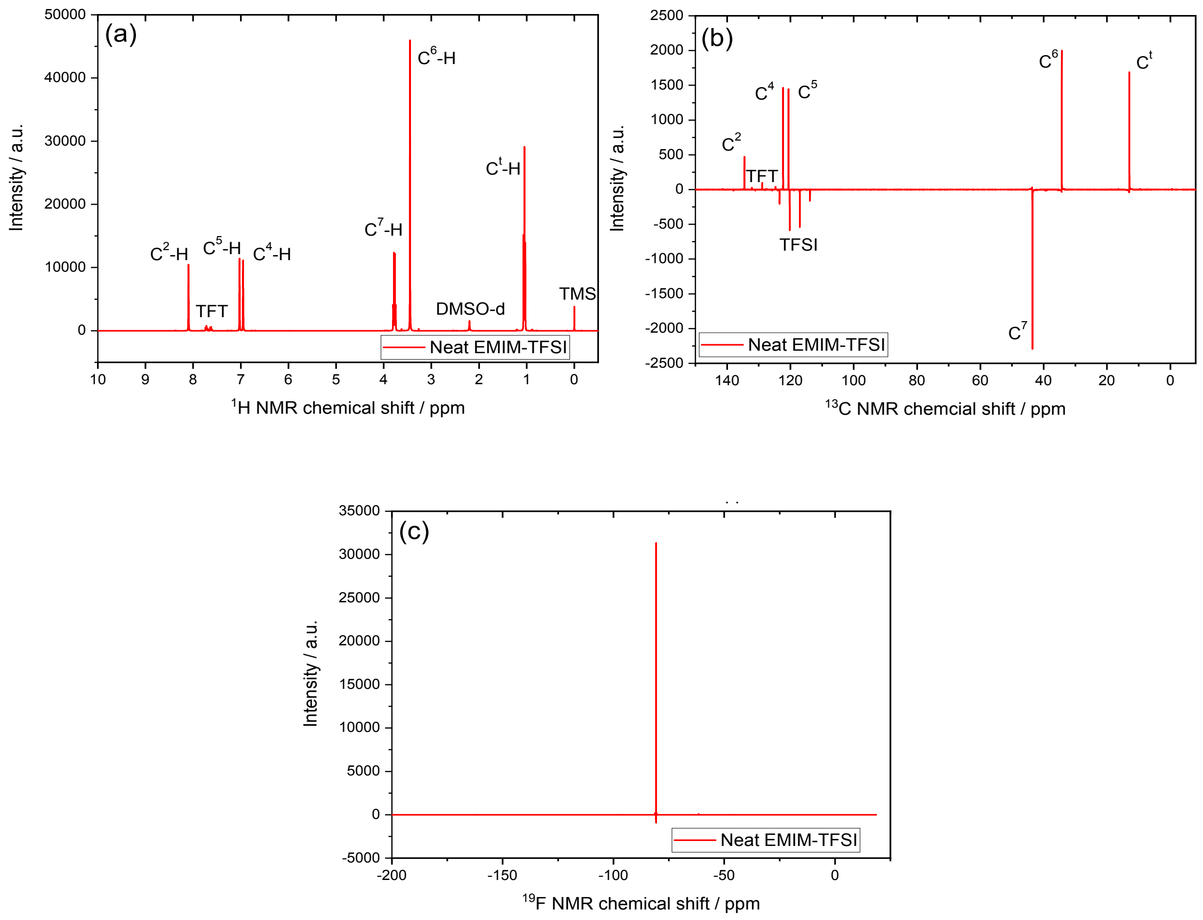

Figure S3: (a) ^{1}H, (b) ^{13}C DEPTQ and (c) ^{19}F NMR spectra of neat EMIM-TFSI.

2. Electrochemical Measurements

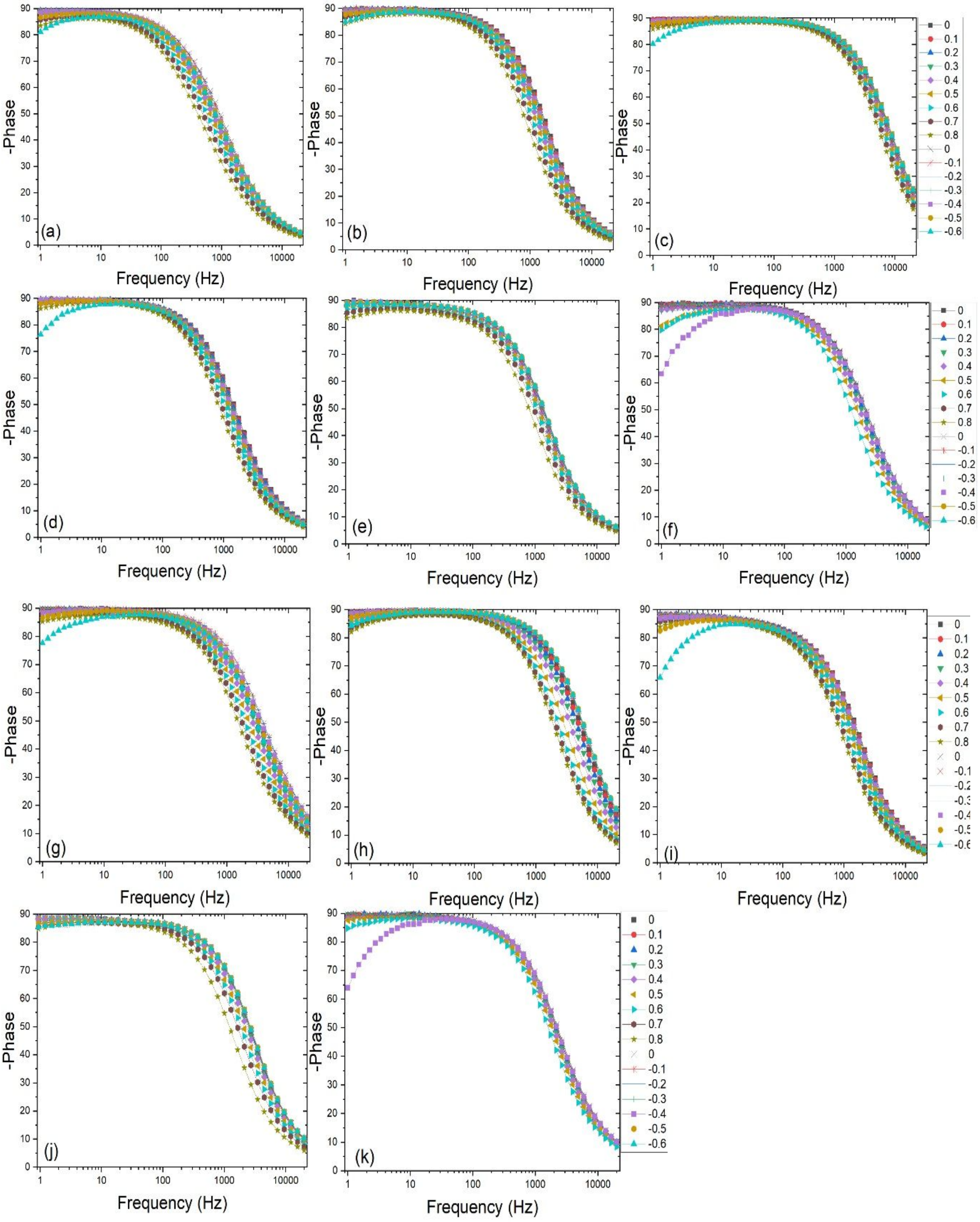

Figure S4: Bode plots of neat EMIM-TFSI, 1M and 2M EMIM-TFSI/solvents on HOPG. (a) Neat EMIM-TFSI, (b)1 M EMIM-TFSI/DEC, (c) 1M EMIM-TFSI/CAN, (d) 1M EMIM-TFSI/DMSO, (e) 1M EMIM-TFSI/PC, (f) 1M EMIM-TFSI/FD, (g) 2M EMIM-TFSI/DEC, (h) 2M EMIM-TFSI/CAN, (i) 2M EMIM-TFSI/DMSO, (j) 2M EMIM-TFSI/PC and (k) 2M EMIM-TFSI/FD.

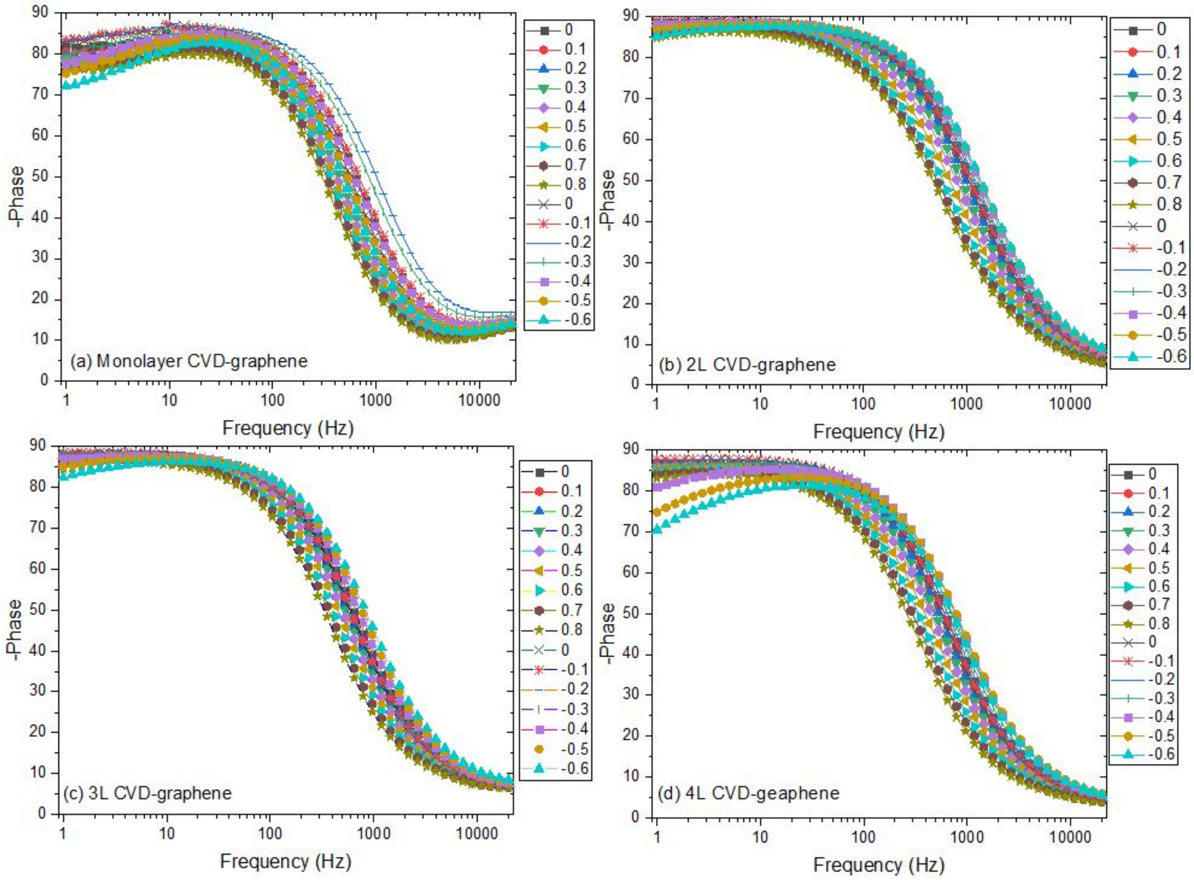

Figure S5: Bode plots of neat EMIM-TFSI at mono- to 4 layers CVD graphene interfaces. (a) Monolayer CVD graphene, (b) 2L CVD graphene, (c) 3L CVD graphene and (d) 4L CVD graphene.

3. ^{13}C and ^{19}F NMR spectra of the EMIM-TFSI mixtures

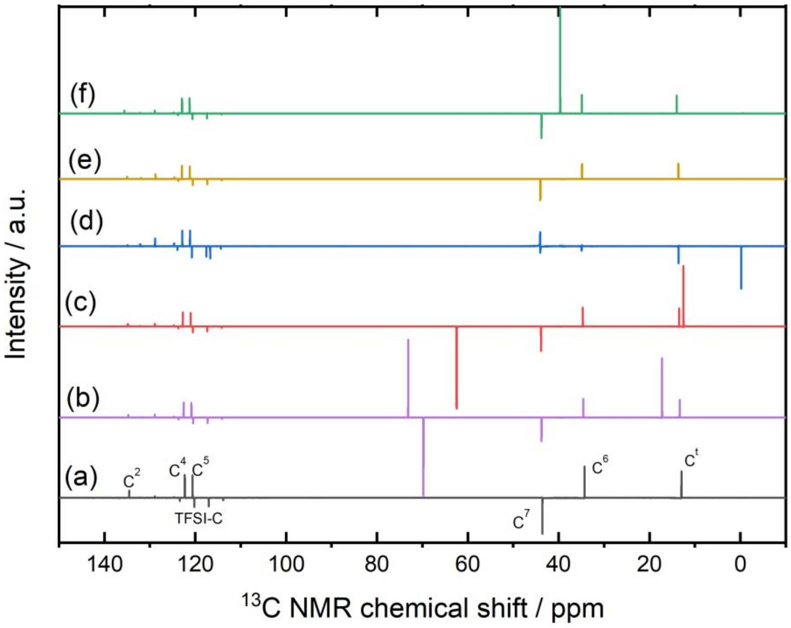

Figure S6: ^{13}C DEPTQ NMR spectra of 2M EMIM-TFSI/solvents mixtures (a) Neat EMIM-TFSI (b) 2M EMIM-TFSI/PC (c) 2M EMIM-TFSI/DEC (d) 2M EMIM-TFSI/ACN (e) 2M EMIM-TFSI/FD (f) 2M EMIM-TFSI/DMSO.

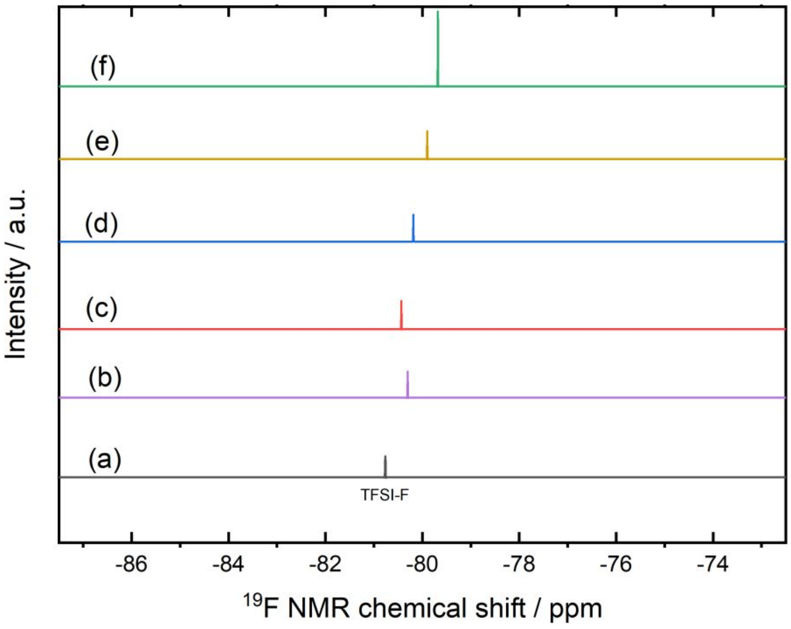

Figure S7: ^{19}F NMR results of 2M EMIM-TFSI/solvents mixtures (a) Neat EMIM-TFSI (b) 2M EMIM-TFSI/PC (c) 2M EMIM-TFSI/DEC (d) 2M EMIM-TFSI/ACN (e) 2M EMIM-TFSI/FD (f) 2M EMIM-TFSI/DMSO.

4. Capacitance data for the EMIM-TFSI/DMSO/H_2O mixtures

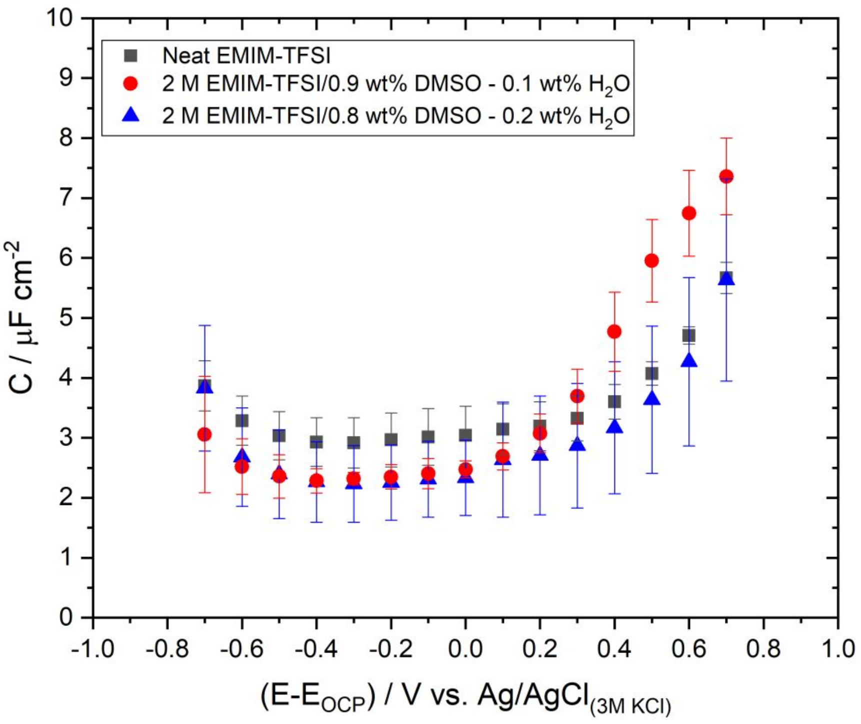

Figure S8: Differential capacitance, C, vs. applied potential, E, plots recorded on the HOPG in contact with 2 M mixtures of EMIM-TFSI with 0.9 wt.% dimethyl sulfoxide (DMSO, ε_r = 47) + 0.1 wt.% and 0.8 wt.% dimethyl sulfoxide (DMSO, ε_r = 47) + 0.2 wt.% following the experimental protocol described in the Experimental section. C_{EDL} was extracted using equation (2) from the EIS data recorded in the capacitive potential window (see Experimental section). For comparison purposes the data presented in Figure 2 corresponding to the neat EMIM-TFSI is also given.

References

(1) D'Agostino, C.; Mantle, M. D.; Mullan, C. L.; Hardacre, C.; Gladden, L. F. Diffusion, Ion Pairing and Aggregation in 1-Ethyl-3-Methylimidazolium-Based Ionic Liquids Studied by 1H and 19F PFG NMR: Effect of Temperature, Anion and Glucose Dissolution. *ChemPhysChem* **2018**, *19* (9), 1081–1088. https://doi.org/10.1002/cphc.201701354.
